# Functional Olfactory Nerve Regeneration Demonstrated by Thallium-201 Olfacto-Scintigraphy in Patients with Traumatic Anosmia: A Case Report

**DOI:** 10.1155/2019/1069741

**Published:** 2019-11-16

**Authors:** Rong-San Jiang, Yu-Yu Lu

**Affiliations:** ^1^Department of Medical Research, Taichung Veterans General Hospital, Taichung, Taiwan; ^2^Department of Otolaryngology, Taichung Veterans General Hospital, Taichung, Taiwan; ^3^School of Medicine, Chung Shan Medical University, Taichung, Taiwan; ^4^Rong Hsing Research Center for Translational Medicine, National Chung Hsing University, Taichung, Taiwan; ^5^Department of Nuclear Medicine, Taichung Veterans General Hospital, Taichung, Taiwan

## Abstract

Head trauma is one of the most common etiologies of olfactory dysfunction. It is difficult to use either the olfactory function test or magnetic resonance imaging to directly assess the course of damage to olfactory nerves. Thallium-201 (^201^Tl) olfacto-scintigraphy has been shown to be an able means for objectively assessing the olfactory nerve transport function. It is expected to be used to evaluate olfactory nerve regeneration after damage to the olfactory nerves. However, no such result has been reported. We present a patient who lost his olfactory function after experiencing head trauma. When his olfactory function remained anosmic, a ^201^Tl olfacto-scintigraphy showed no migration of ^201^Tl from the nasal mucosa to the olfactory bulb. After treatment with medicines and olfactory training, his olfactory function improved. A second ^201^Tl olfacto-scintigraphy showed an increased migration of ^201^Tl from the nasal mucosa to the olfactory bulb.

## 1. Introduction

Head trauma is one of the most common etiologies of olfactory dysfunction [1]. Posttraumatic olfactory dysfunction has been assumed to result from the shearing of olfactory fibers at the cribriform plate, mechanical nasal obstruction, or central brain trauma [2]. Although recovery of olfactory function has been observed in a portion of posttraumatic patients either spontaneously or after treatment [3–6], it is difficult to use the olfactory function test or magnetic resonance imaging (MRI) to directly assess the connectivity of the olfactory nerve to the olfactory bulb [7].

Thallium-201 (^201^Tl) olfacto-scintigraphy has been put into use by Japanese doctors [8]. Thallium ion was shown to be taken up into the olfactory receptor cells from the nasal mucosa and transported via the olfactory nerve to the olfactory bulb [9, 10]. Therefore, it is considered that ^201^Tl olfacto-scintigraphy is a potential clinical imaging tool for the objective assessment of the olfactory nerve transport function [9]. Using ^201^Tl olfacto-scintigraphy, the migration of ^201^Tl from the nasal mucosa to the olfactory bulb was found to be significantly lower in patients with olfactory dysfunction than that of healthy subjects [7]. On the contrary, whether an increase in ^201^Tl migration to the olfactory bulb is correlated with an improvement in the olfactory function in patients with olfactory dysfunction remains helpful for assessing olfactory nerve regeneration after damage of the olfactory nerves [7]. To date, no follow-up data regarding ^201^Tl olfacto-scintigraphy has been reported in patients with olfactory dysfunction.

Herein, we report on a patient who lost his olfactory function after head trauma. When his olfactory function remained anosmic, a ^201^Tl olfacto-scintigraphy showed no migration of ^201^Tl from the nasal mucosa to the olfactory bulb. After treatment with medicines and receiving olfactory training, the patient's olfactory function improved. A second ^201^Tl olfacto-scintigraphy showed increased migration of ^201^Tl from the nasal mucosa to the olfactory bulb. This is the first case to objectively evaluate olfactory nerve regeneration using ^201^Tl olfacto-scintigraphy.

## 2. Case Presentation

**Figure 1:**
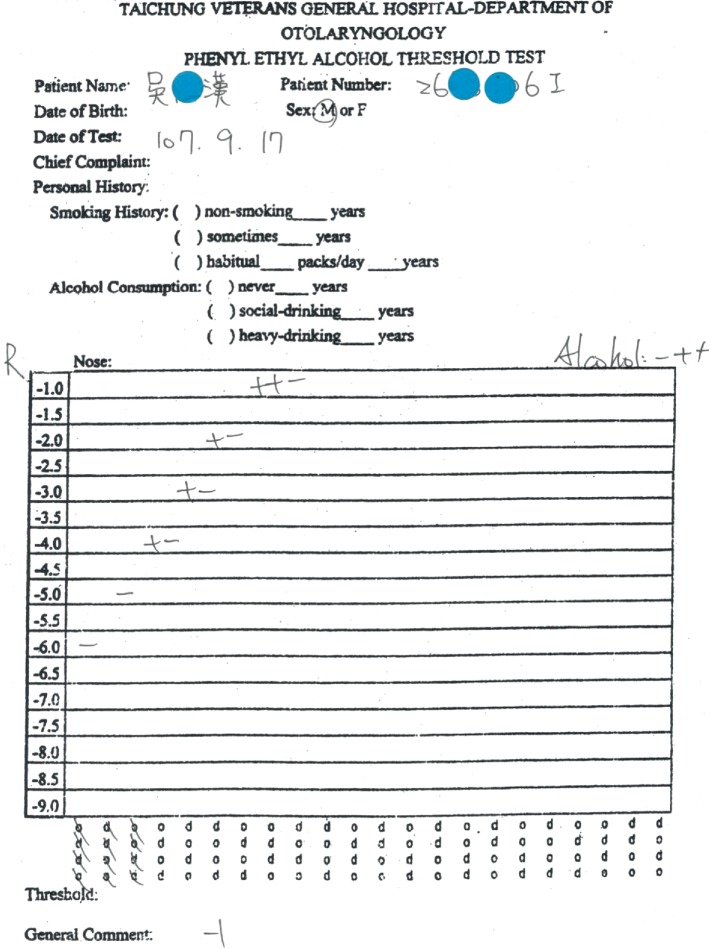
The right unirhinal PEA threshold was −1.

**Figure 2:**
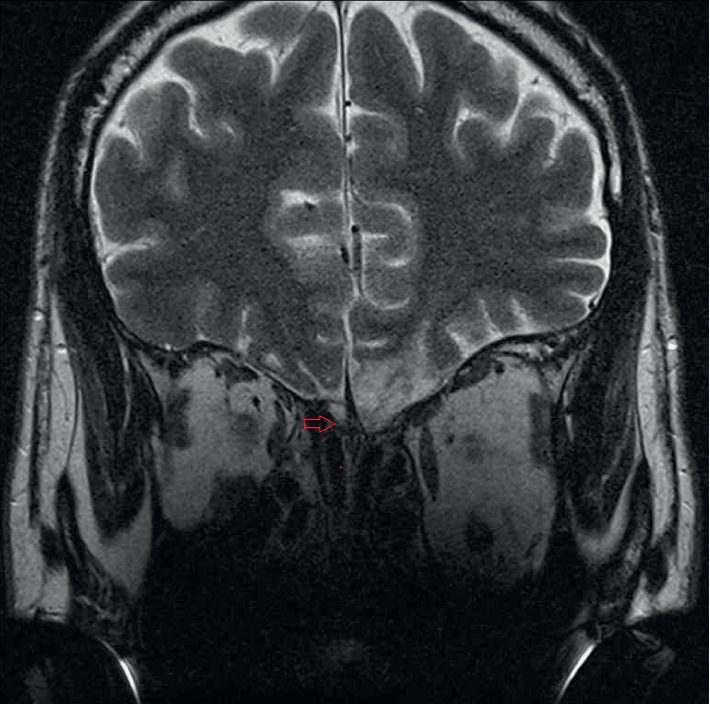
MRI shows irregular hyperintensity over bilateral rectus gyri, with extensive tissue loss at the left frontal base. The sizes of the right olfactory bulb (red arrow) and tract were relatively small.

A 39-year-old male came to our clinic with a complaint of having experienced a loss of olfactory function after a traffic accident on January 22, 2018. At that time, he had been admitted to another hospital where an intracranial hemorrhage was diagnosed through the use of computed tomography. During his first visit to our clinic, a scar was discovered on the left side of his forehead. He received the phenyl ethyl alcohol (PEA) odor detection threshold test, and his birhinal olfactory threshold was determined to be −1. He was placed on a course of high-dose prednisolone (1 mg/kg per day) with tapering for 2 weeks, along with a month-long zinc gluconate course (10 mg/3 times per day), followed by 6-month olfactory training involving the 4 traditional odorants (PEA, lemon, eucalyptus, and clove oils). After treatment, his birhinal and unirhinal PEA thresholds had remained at −1 (Figure 1). An MRI showed irregular hyperintensity over the bilateral rectus gyri, along with extensive tissue loss at the left frontal base. The sizes of the right olfactory bulb and tract were relatively small (Figure 2).

A ^201^Tl olfacto-scintigraphy was arranged in order to evaluate the right olfactory nerve connectivity. The procedures for the ^201^Tl olfacto-scintigraphy followed those used by Shiga et al. [10]. The patient lay down on a bed, where 0.3 mL of ^201^Tl saline solution (22 MBq) was instilled into the right olfactory cleft using a syringe under a nasal endoscope. The patient then turned his body to the left to be in the lateral decubitus position for 30 minutes.

**Figure 3:**
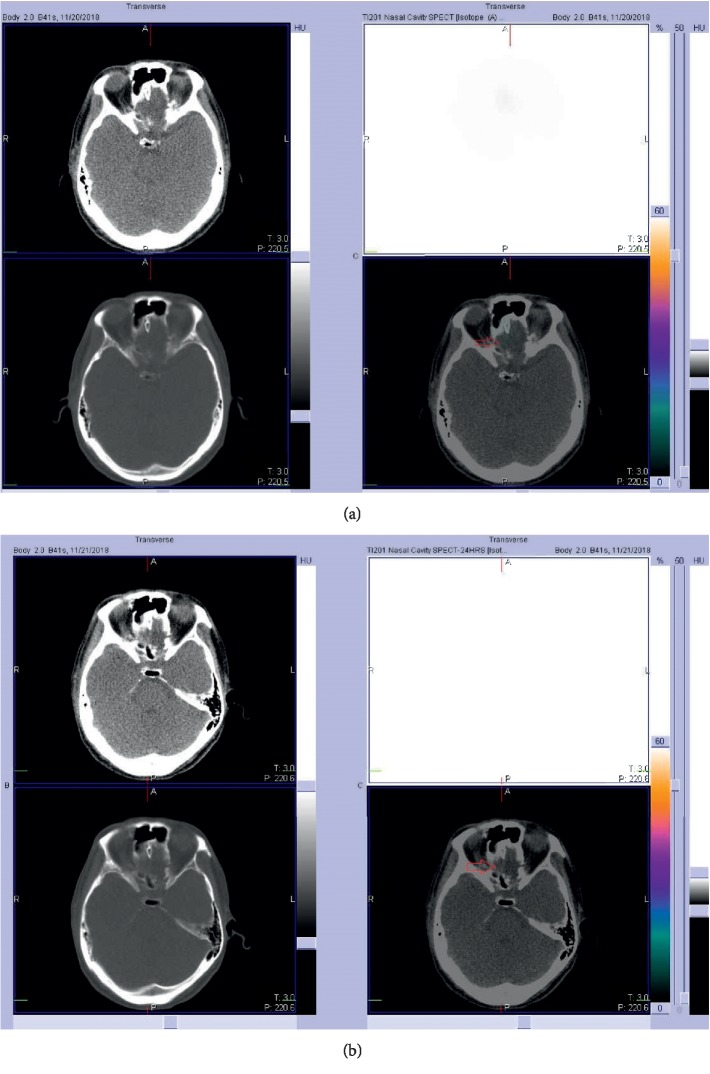
(a) No tracer accumulated at the right olfactory bulb (red arrow) on the 30 minute images. (b) No tracer accumulated at the right olfactory bulb (red arrow) on the 24 hour images.

Localizing planar and hybrid SPECT/CT images taken overhead were acquired both 30 minutes and 24 hours after ^201^Tl nasal administration. The data acquisition was conducted using a dual-headed SPECT-CT hybrid system (Symbia T; Siemens Medical Solutions, USA), equipped with low-energy high-resolution collimators. Data were acquired in a 128 × 128 matrix with a 2.29 times zoom, through a 360° rotation at a 4° interval, for 30 seconds per projection, along with a 72 keV photopeak with a 30% window and 166 keV photopeak with a 14% window. Data reconstruction was performed with nine subsets and eight iterations. Scatter correction was applied using Flash 3D. The acquisition parameters for CT were as follows: 130 keV, pitch 2.0, and slice thickness 2.0 mm. The acquisition time of SPECT/CT was 30 minutes. The tracer did not accumulate at the right olfactory bulb on the 30 minute images, as well as the 24 hour images (Figure 3).

**Figure 4:**
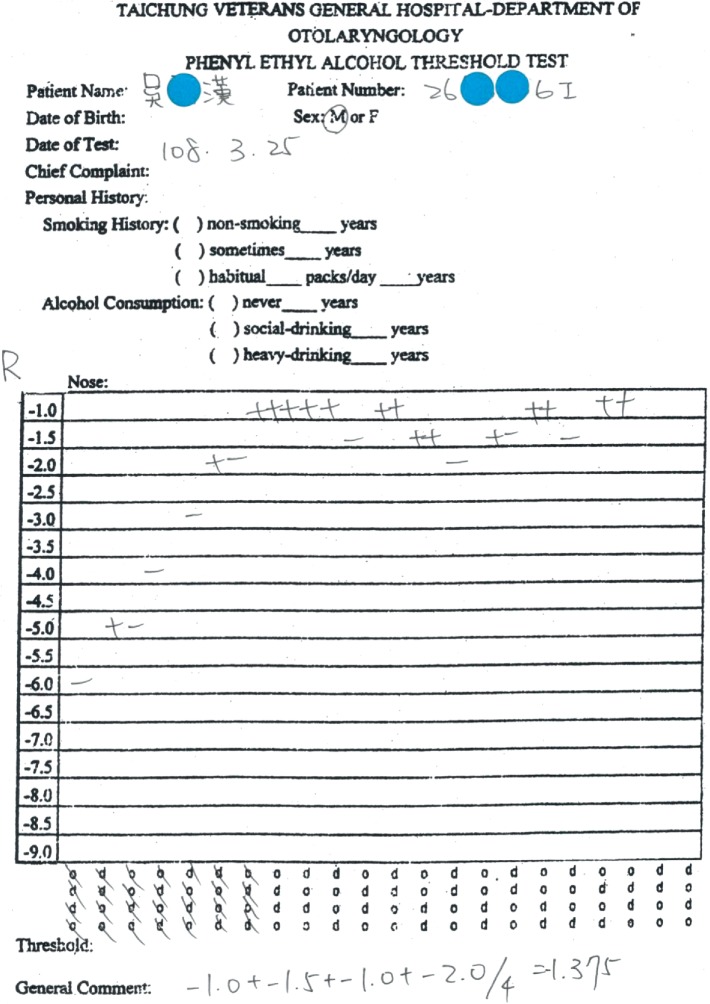
The right unirhinal PEA threshold was −1.375.

**Figure 5:**
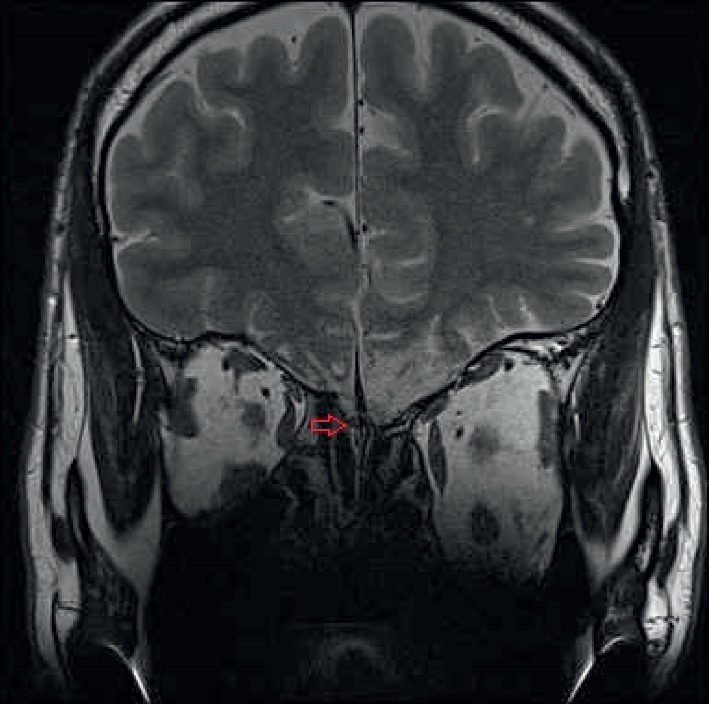
MRI shows that the right olfactory bulb (red arrow) became larger.

**Figure 6:**
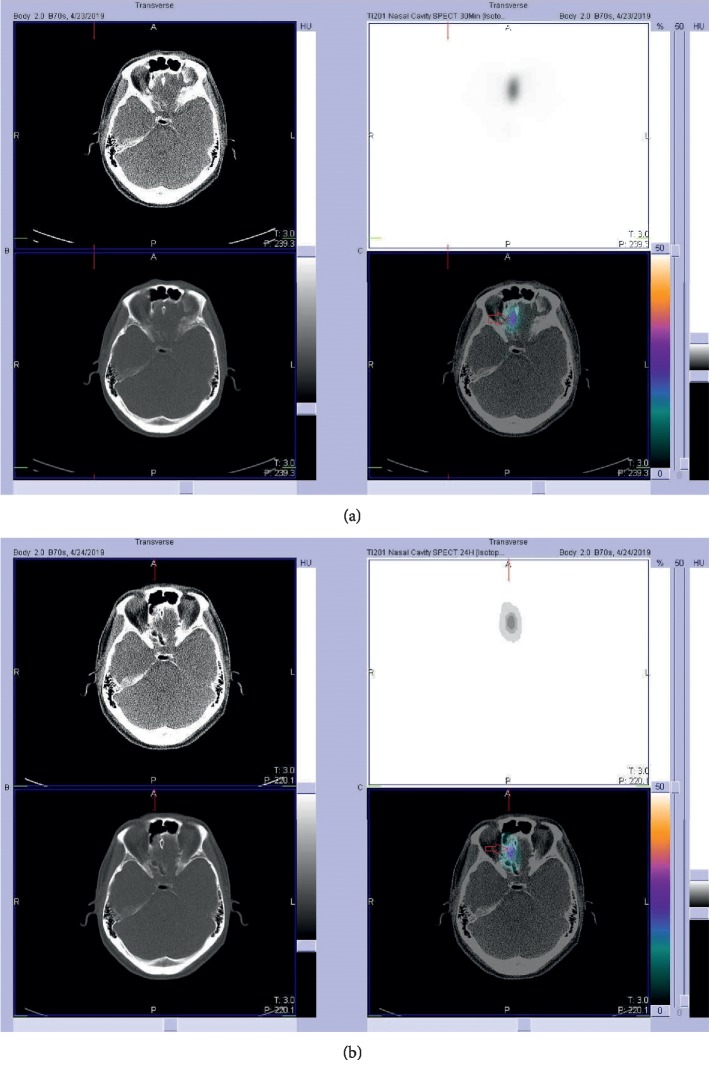
(a) Increased tracer accumulated at the right olfactory bulb (red arrow) on the 30 minute images. (b) Increased tracer accumulated at the right olfactory bulb (red arrow) on the 24 hour images.

Subsequently, the patient was given oral theophylline (200 mg q12h) and kept on olfactory training. Five months later, he reported that his olfactory function had improved, while his birhinal PEA threshold had decreased to −1.75 and his right unirhinal threshold had decreased to −1.75, but his left unirhinal threshold remained at −1. One month later, his birhinal PEA threshold was −3.625 and right unirhinal threshold was −1.35, but left unirhinal threshold remained at −1 (Figure 4). Another MRI was arranged to evaluate the right frontal bulb, and a ^201^Tl olfacto-scintigraphy was scheduled to assess the right olfactory nerve connectivity. The MRI revealed a relatively large right olfactory bulb, as compared with the previous MRI (Figure 5). The ^201^Tl olfacto-scintigraphy demonstrated increased trace accumulation at the right frontal bulb on both the 30 minute and 24 hour images (Figure 6).

## 3. Discussion

^201^Tl has been widely employed through the use of intravenous injection in isotope imaging for myocardial and tumor scanning [11, 12]. The biological safety regarding the use of ^201^Tl olfacto-scintigraphy has been established in both animals and humans [10, 12]. Most intranasally administered ^201^Tl migrates to the area of the nasopharynx and is swallowed. Since ^201^Tl is rarely absorbed by the central nervous system, the potential systemic effects of the swallowed ^201^Tl are able to be ignored [7].

^201^Tl olfacto-scintigraphy has been used to evaluate the olfactory nerve transport function [10]. The migration of ^201^Tl to the olfactory bulb was found to be significantly lower in patients with olfactory dysfunction than that in healthy subjects, with the ratio of migration being correlated to the olfactory function and the volume of the olfactory bulb [7]. It is difficult to predict the prognosis in patients with posttraumatic olfactory dysfunction [13]. Recently, it has been shown that a higher migration of ^201^Tl to the olfactory bulb was significantly correlated with a better prognosis in patients with olfactory dysfunction [8]. It has been further proposed that an increase in the migration of ^201^Tl to the olfactory bulb during treatment may be correlated with an improvement in olfactory function, but no such data has yet been reported [7].

In our patient, an MRI showed olfactory bulb damage which was more severe in the left side upon experiencing head trauma. His olfactory function was anosmic. Although he received 7 months of treatment, he remained anosmic. When a ^201^Tl olfacto-scintigraphy was arranged to evaluate the right olfactory nerve connectivity, no tracer had accumulated at the right olfactory bulb on the 30 minutes, as well as on 24 hour images. This indicated that the olfactory nerve did not connect to the olfactory bulb. After the patient's treatment continued for another 5 months, his right PEA threshold decreased. An MRI was arranged to reevaluate the olfactory bulbs, and the right olfactory bulb became more visible. A ^201^Tl olfacto-scintigraphy was arranged to reevaluate the right olfactory nerve connectivity, and an increased trace accumulation was seen at the right frontal bulb on both the 30 minute and 24 hour images. This is the first report to demonstrate the regeneration of the olfactory nerve during treatment in a patient with posttraumatic olfactory dysfunction using ^201^Tl olfacto-scintigraphy. In the future, additional patients will be gathered to better study the role of ^201^Tl olfacto-scintigraphy when exploring the relationship between olfactory function and olfactory nerve connectivity.
